# Mixed strain pathogen populations accelerate the evolution of antibiotic resistance in patients

**DOI:** 10.1038/s41467-023-39416-2

**Published:** 2023-07-12

**Authors:** Julio Diaz Caballero, Rachel M. Wheatley, Natalia Kapel, Carla López-Causapé, Thomas Van der Schalk, Angus Quinn, Liam P. Shaw, Lois Ogunlana, Claudia Recanatini, Basil Britto Xavier, Leen Timbermont, Jan Kluytmans, Alexey Ruzin, Mark Esser, Surbhi Malhotra-Kumar, Antonio Oliver, R. Craig MacLean

**Affiliations:** 1 University of Oxford, Department of Biology, 11a Mansfield Rd, Oxford, UK; 2 Servicio de Microbiología, Hospital Universitari Son Espases, Instituto de Investigación Sanitaria Illes Balears (IdISBa), Palma de Mallorca, Spain; 3 Laboratory of Medical Microbiology, Vaccine and Infectious Disease Institute, University of Antwerp, Wilrijk, Belgium; 4 Julius Center for Health Sciences and Primary Care, University Medical Center Utrecht, Utrecht University, Utrecht, The Netherlands; 5 Microbial Sciences, BioPharmaceuticals R&D, AstraZeneca, Gaithersburg, MD USA

**Keywords:** Antimicrobial resistance, Experimental evolution

## Abstract

Antibiotic resistance poses a global health threat, but the within-host drivers of resistance remain poorly understood. Pathogen populations are often assumed to be clonal within hosts, and resistance is thought to emerge due to selection for de novo variants. Here we show that mixed strain populations are common in the opportunistic pathogen *P. aeruginosa*. Crucially, resistance evolves rapidly in patients colonized by multiple strains through selection for pre-existing resistant strains. In contrast, resistance evolves sporadically in patients colonized by single strains due to selection for novel resistance mutations. However, strong trade-offs between resistance and growth rate occur in mixed strain populations, suggesting that within-host diversity can also drive the loss of resistance in the absence of antibiotic treatment. In summary, we show that the within-host diversity of pathogen populations plays a key role in shaping the emergence of resistance in response to treatment.

## Abstract (web summary)

Here, Caballero et al. provide an in depth characterisation of patients colonized with single or mixed strains of *Pseudomonas aeruginosa* to demonstrate the impact of within-host diversity on the development of antibiotic resistance.

## Introduction

Antibiotic resistance in pathogenic bacteria poses a fundamental threat to human health. It is well established that antibiotic use is associated with the emergence of resistance^1,2^. However, the within-host drivers of resistance remain poorly understood, making it difficult to predict the emergence of resistance at the scale of individual patients^3–5^. This is an important problem to address, as resistant infections are associated with worse outcomes for patients^6,7^.

The dominant model for the within-host emergence of resistance is that resistance evolves because of selection for novel alleles that are acquired in situ by mutation or horizontal gene transfer^4,8–12^. An implicit assumption of this model is that hosts are colonized by clonal pathogen populations that lack genetic variation due to bottlenecks that occur during transmission^8,13–16^. However, hosts can also be colonized by multiple strains of the same pathogen species^17–22^. Mixed strain populations contain both novel genetic variation that is acquired in situ and pre-existing variation that reflects differences between the co-colonizing strains. A key concept from evolutionary biology is that this additional source of standing genetic variation in mixed strain populations should accelerate the evolutionary response to antibiotic treatment by increasing the genetic diversity that selection acts on^23–25^. This simple logic predicts that resistance will evolve rapidly in hosts colonized by diverse pathogen populations.

In this paper, we test the hypothesis that resistance evolves rapidly in diverse populations of *Pseudomonas aeruginosa*, an opportunistic pathogen that is an important cause of hospital-acquired infection, particularly in immunocompromised and critically ill patients^7,26–28^. *Pseudomonas* can cause infections at a wide range of anatomical sites, but the biggest problem with *Pseudomonas* comes from infections of the lung^7,28–30^. *P. aeruginosa* evolves resistance during infections at a very high rate compared to other ESKAPE pathogens^31^, and resistance is an importance challenge for treating *Pseudomonas* infection^7,31^. Previous studies have shown that different strains of *Pseudomonas* can co-occur in the lungs of patients who suffer from chronic *P. aeruginosa* infection associated with cystic fibrosis and bronchiectasis^21,22,32^. We tested the impact of within-patient *Pseudomonas* diversity in ICU patients using samples that were collected from ASPIRE-ICU, an observational trial of *Pseudomonas* infection in European hospitals^33^.

Patients who were enroled into the ASPIRE-ICU trial were screened for *Pseudomonas* soon after admission to ICU and then at regular intervals thereafter. Clinical microbiology projects usually focus on the analysis of a small number of isolates per patient sample, making it difficult to assess the prevalence and importance of within-patient pathogen diversity. ASPIRE-ICU, on the other hand, sampled *Pseudomonas* isolates in an unbiased manner (i.e. without respect to resistance phenotype), and collected up to 12 randomly chosen isolates from all patient samples containing *Pseudomonas*. Most patients who were enroled in the trial spent only a short time in ICU, but longitudinal samples were collected from some patients, making it possible to directly study the within-patient drivers of antibiotic resistance^8,34^. In this paper, we use a combination of phenotypic assays (resistance phenotyping, growth rate) and genomic analyses to quantify within-patient diversity and antibiotic resistance. Using this approach we show that at least 1/3 of patients are colonized by multiple *Pseudomonas* strains, and that resistance evolves rapidly in these patients due to selection for pre-existing resistant strains.

## Results

### Genomic diversity of *Pseudomonas aeruginosa*

**Fig. 1:**
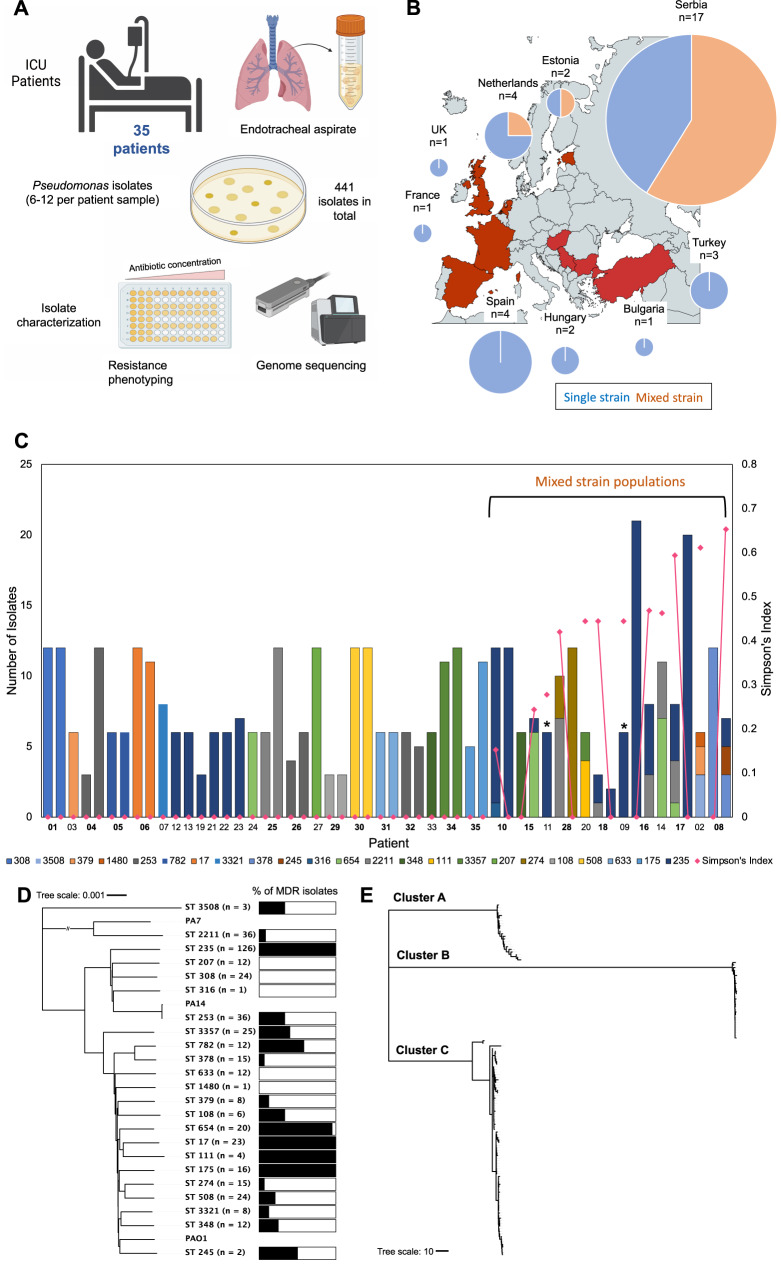
Overview of patient cohort, methodology, and isolate dataset. **A** Overview of sample collection. Endotracheal aspirate was collected from 35 patients with up to 12 *Pseudomonas* isolates per patient sample. A total of 441 isolates were collected and characterized via resistance phenotyping and genome sequencing. **B** Map showing distribution of patient samples. Pie charts show total mixed strain (orange) and single strain (blue) patients per country. Pie chart size is adjusted by patient number, patient number is shown by ‘n=’ above the corresponding chart. Figure was created using mapchart.net. **C** Within-host diversity of *P. aeruginosa*. Bar height indicates the number of isolates and bar colour indicates the Sequence Type(s) (ST) of *P. aeruginosa* collected from each patient in the study cohort. Mixed strain populations were identified in 12/35 patients. Patient numbers in bold indicate longitudinally sampled patients, and the isolates from each sample are shown sequentially. * indicates patients with a mixed strain population consisting of isolates from multiple distinct ST235 sub-lineages. Plotted points show the diversity of clones within patients, as measured by Simpson’s Index, and the lines show how Simpson’s Index changes in between samples in patients with two samples. **D** Neighbour joining phylogeny based on core genes of all STs found in this study and the respective proportion of MDR isolates in each ST^82^. nucleotide substitutions per site. **E** Neighbour joining phylogeny based on core genes of ST235 isolates showing the three distinct ST235 sub-lineages (cluster A, B and C)^82^. Scale = nucleotide substitutions. The raw data is presented in the source data file.

To characterize the diversity of *P. aeruginosa* within patients we sequenced the genomes of 441 isolates that were collected from lower respiratory tract samples of 35 ICU patients from 12 hospitals (Fig. 1A). The number of patients per hospital was very unevenly distributed, and ~50% (*n* = 17/35 patients) of our patients were from a single hospital with a high *Pseudomonas* colonization rate (Fig. 1B) (Source data).

In line with previous work^35^, we found that *P. aeruginosa* has a non-epidemic clonal population structure, consisting of clearly differentiated Sequence Types (STs) that are separated by long branches (Fig. 1D). The most prevalent ST (ST235) segregated into three sub-lineages, which diversified prior to our sampling (Fig. 1E). Given this phylogeny, we considered STs and sub-lineages of ST235 to represent unique strains.

The majority of patients (*n* = 23/35) were colonized by a single strain (Fig. 1C). However, ~1/3 of patients (*n* = 12/35) were colonized by multiple strains (Fig. 1C). Most of the patients that were colonized by multiple strains were from a single hospital with a high *Pseudomonas* colonization rate (Fig. 1B). However, the uneven sampling of patients across hospitals made it impossible to test for variation in the prevalence of mixed strain colonization across hospitals (Fig. 1B). Previous work has found evidence of mixed strain populations in chronic *Pseudomonas* infections^21,32^, suggesting that mixed strain populations may be found in patients with a long history of *Pseudomonas* infection. However, the prevalence of mixed strain populations did not differ between patients who tested positive for *Pseudomonas* on ICU admission versus those who were colonized in ICU, suggesting that high within-patient diversity is not simply a consequence of chronic infection (unpaired two-tailed *t*-test; *p* = 0.9073) (Supplementary Table 1).

Strain diversity tended to be high in patients colonized by multiple strains (mean Simpson’s index = 0.37, st.dev = 0.15) (Fig. 1C). This measure of within-patient diversity does not depend on the number of isolates sampled, but our estimate of the prevalence of mixed strain colonization should be treated as conservative, as our ability to detect rare strains was limited by the number of isolates sequenced per patient. For example, in patients with six sequenced isolates, the probability of detecting a rare strain present at a frequency of 5% is only 26%, assuming a binomial sampling distribution. However, in patients with >10 isolates, the probability of detecting a rare strain increases to above 40%. We carried out rarefaction analysis of the mixed strain patient populations to estimate the power of our sampling to detect mixed strain populations. This showed that with six isolates per sample (which is the median isolate number in our single strain patient samples), multiple strains are captured in >99% of the subsamples for 8/9 mixed strain patients (Supplementary Fig. 1).

### Within-patient diversity and AMR

To understand the impact of within-patient diversity on antimicrobial resistance (AMR), we measured resistance of isolates to a panel of antibiotics including ciprofloxacin, meropenem, gentamicin, aztreonam, ceftazidime and piperacillin/tazobactam using minimum inhibitory concentration (MIC) assays. This panel included representatives of all of the major families of antibiotics, but was biased towards β-lactam antibiotics due to their clinical relevance for treating *Pseudomonas* infections^36^. In total we measured the resistance of 441 isolates to six antibiotics (Fig. 1A), generating a large dataset on the distribution of resistance phenotypes (Source data).

Measuring antibiotic resistance using MIC assays generates quantitative data on resistance. However, we chose to analyse the resistance of each isolate to each antibiotic as a binary trait (i.e. sensitive/resistant) for two reasons. First, the selective impact of quantitative variation in MIC scores that are above the clinical breakpoint concentration is not clear, as pathogens may not encounter these high antibiotic doses during treatment^37^. Furthermore, some bacterial isolates were resistant to the maximal doses of antibiotic used in our susceptibility assays, and in these cases the quantitative resistance score is undefined.

**Fig. 2:**
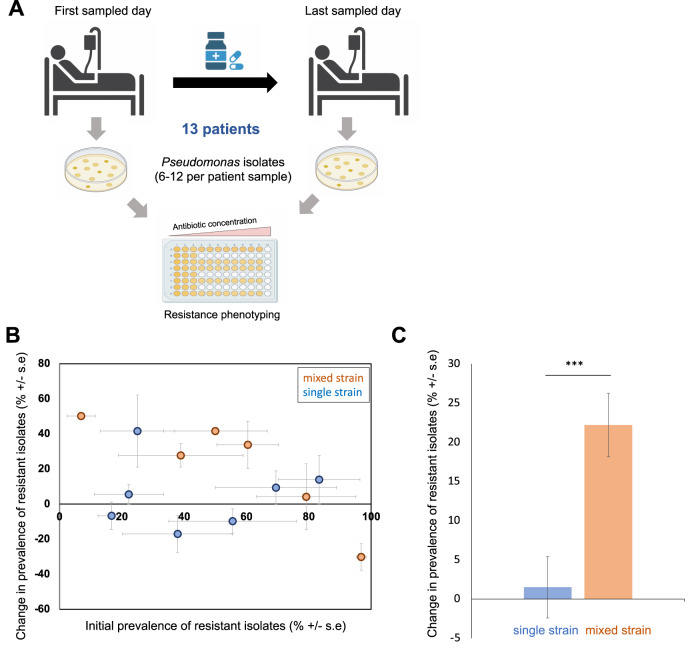
Mixed strain populations accelerate the emergence of resistance. **A** Resistance phenotyping of isolates from the subset of 13 longitudinally sampled patients that were treated with antibiotics that are active against *Pseudomonas*. **B** Change in the prevalence of antibiotic-resistant isolates in patients with single strain (blue) and mixed strain (orange) populations. Resistance to six antibiotics was measured and the proportion of isolates that were resistant to each antibiotic at each time point was calculated. Data are presented as mean values for the proportion of isolates that were resistant to each antibiotic for the first sampling point (initial prevalence) and the difference between sampling points (change in prevalence), from a minimum of *n* = 5 isolates per patient (source data file). Error bars show the standard error of initial resistance (x-axis; *n* = 6 antibiotics) and the change in resistance (y-axis; *n* = 6 antibiotics) across antibiotics. **C** Bars show the mean (+/−s.e.) change in the prevalence of resistant isolates in single strain (*n* = 7 patients; blue) and mixed strain (*n* = 6 patients; orange) populations after correcting for the effect of initial resistance. Mixed strain populations were associated with larger increases in resistance in response to antibiotic treatment (main effect diversity, F_1,52_ = 15.03, *P* = 0.0003). The statistical model used to analyse this data is described in the methods and the model output is given in Supplementary Table 3. The raw data is presented in the source data file.

For approximately half the patients (*n* = 16/35) we obtained isolates from only a single patient sample. Isolates from these patients provide insights into within-host diversity, but they provided limited insights into evolutionary drivers of AMR. To address this problem, we measured changes in resistance using isolates from samples that were collected from patients before and after treatment with antibiotics that are active against *P. aeruginosa* (Fig. 2A). This subset of 13 longitudinally sampled patients included patients with single strain (*n* = 7 patients) and mixed-strain (*n* = 6 patients) *Pseudomonas* populations. The remaining 6 longitudinally sampled patients were not treated with antibiotics that are active against *P. aeruginosa* between their sampling points. Importantly, the number of days between the initial and final sample did not differ between patients with single strain and mixed strain populations (unpaired two-tailed t-test; *p* = 0.3211) (Supplementary Table 2).

To measure the response to antibiotic treatment, we calculated the change in the proportion of isolates that were resistant to each antibiotic over time for each combination of patient and antibiotic. Measuring resistance to a panel of antibiotics allowed us to distinguish between direct responses to antibiotics that were used in treatment and collateral changes in resistance to antibiotics that were not used for treatment for each patient (Source data). Surprisingly, direct responses to antibiotic treatment were not larger than collateral responses, implying an overall tendency towards cross-resistance (main effect response type: F_1,52_ = 0.029, *P* = 0.86) (Supplementary Table 3). Given this, our analysis of changes in resistance in response to treatment included all antibiotics in our panel. An important implication of this approach is that we assumed that each combination of patient and antibiotic was an independent response.

Antibiotics should impose strong selection for resistance in populations where average levels of resistance are low. Consistent with this hypothesis, resistance increased rapidly in patients where the initial prevalence of resistant isolates was low (Fig. 2B; main effect initial resistance, F_1,52_ = 32, *P* < 0.0001). Importantly, this effect of initial resistance did not depend on the diversity of the pathogen population (initial resistance*diversity interaction: F_1,52_ = 0.018, *P* = 0.894) (Supplementary Table 3). Response to antibiotic treatment varied between patients, even after correcting for the impact of initial resistance, implying that individual combinations of host/pathogen/treatment played an important role in the evolution of resistance (nested effect of patient: F_11,52_ = 3.91, *P* = 0.0004) (Supplementary Table 3). Crucially, increases in resistance were ~20% larger in patients colonized by mixed strains compared to single strains for any given level of initial resistance (Fig. 2C; main effect diversity, F_1,52_ = 15.03, *P* = 0.0003) (Supplementary Table 3).

### Drivers of resistance in mixed strain infections

The emergence of resistance in patients with mixed strain populations could be caused by either selection for pre-existing resistant strains or selection for novel variants. To test for selection on pre-existing strains, we simplified our antibiotic resistance phenotyping data by classifying each isolate as multi-drug resistant (MDR; resistant to 3 or more antibiotics) or non-MDR (Source data). This is an appropriate measure of resistance due to the overall tendency towards cross-resistance, and using this metric simplifies subsequent analysis.

**Fig. 3:**
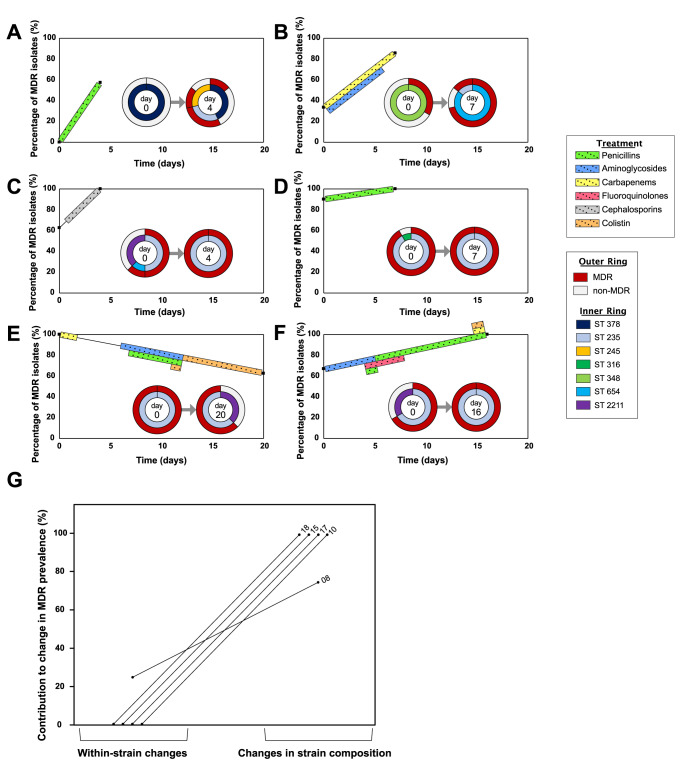
Resistance emerges due to selection for pre-existing resistant strains. **A**–**F** Changes in the prevalence of MDR isolates (resistant to 3 or more antibiotics) and strain composition in longitudinally sampled patients with mixed strain populations. Percentage of MDR isolates is shown between the first and last sampling points. Colour blocks between these sampling points indicate patient antibiotic use. The inset pie charts show the proportion of STs at each time point (inner ring), and the contribution of these STs to MDR (outer ring). Each inset pie chart is labelled with the day of sampling it corresponds to. **A** Patient 8, **B** Patient 15, **C** Patient 17, **D** Patient 10, **E** Patient 16, **F** Patient 18. **G** For these five mixed strain population patients where resistance increases, we partitioned the increases in the prevalence of MDR isolates into changes in ST composition and changes in multi-drug resistance within strains. Numbers indicate patient ID. The raw data is presented in the source data file.

Examining changes in the composition of mixed strain populations revealed that STs that were associated with non-MDR isolates were repeatedly replaced by ST235 and ST654 (Fig. 3A–F). Both of these strains are well-characterized and epidemiologically successful MDR strains of *P. aeruginosa*^38^ that are common in hospitals from south-eastern Europe^39^. In 3/5 patients where resistance increased in response to treatment, the resistant strain was already detected prior to treatment (Fig. 3A, C, D). In patient 15 (Fig. 3B), the ST654 resistant strain that was detected after antibiotic treatment was not sampled at the initial time point. However, polymorphisms were found in the ST654 isolates that were sampled after antibiotic treatment (Supplementary Fig. 2). Given the short time interval between sampling (7 days) and the low rate of within-host evolution of *P. aeruginosa* in critically ill patients (10–20 SNPs/genome/year^8,34^), these pre-existing polymorphisms provide good evidence that ST654 was present at a low frequency prior to antibiotic treatment. In the remaining patient (Patient 8; Fig. 3F), 2 new ST235 MDR strains were detected after antibiotic treatment. The low number of isolates of each of these strains (*n* = 2) made it difficult to test whether these strains were present prior to antibiotic treatment. However, removing this patient from the analysis did not alter any of our conclusions (Supplementary Table 3).

In summary, antibiotic treatment of patients with mixed strain populations was clearly associated with selection for pre-existing resistant strains. One way to quantify this effect is to decompose the increase in the prevalence of MDR isolates in the 5 patients where resistance increased into changes in ST composition, which we assume to reflect pre-existing variation, and changes in the prevalence of MDR within strains, which we assume reflect the impact of new variants that were acquired during infection (Fig. 3G). In 4/5 patients all of the increase in resistance was driven by changes in ST composition, and in the remaining patient changes in ST composition accounted for close to 80% of the increase in MDR (Fig. 3G). Combining these data we estimate that >90% of increase in MDR in patients with mixed strain populations are driven by selection for pre-existing resistant strains. This very clear result suggests that pathogen diversity is associated with the rapid emergence of resistance because diverse pathogen population are more likely to contain pre-existing resistant strains, and not because diversity per se accelerates the emergence of resistance within strains.

### Testing for de novo resistance evolution

To directly estimate the impact of strain diversity on the de novo evolution of resistance, we tested for signatures of resistance acquisition using genomic data from longitudinally sampled patients. The genetic basis of antimicrobial resistance in *Pseudomonas aeruginosa* is complex and multifactorial^40–44^. Most clinically relevant resistance mutations increase the expression of chromosomal resistance genes, such as mutations in peptidoglycan biosynthesis genes *(ampD*, *ampDh2, dab*, *mpl*) that increase the expression of the chromosomal *ampC* β-lactamase, or *nalD* and *mexR* mutations that increase the expression of the MexAB-OprM and MexXY-Z multidrug efflux pumps, respectively. Chromosomal mutations can also confer increased resistance by altering antibiotic targets, such as *gyrA* and *parC* mutations that modify fluoroquinolone target sites. Finally, mutations can restrict the entry of antibiotics into the cell, such as *oprD* mutations that provide resistance to carbapenem antibiotics.

**Fig. 4:**
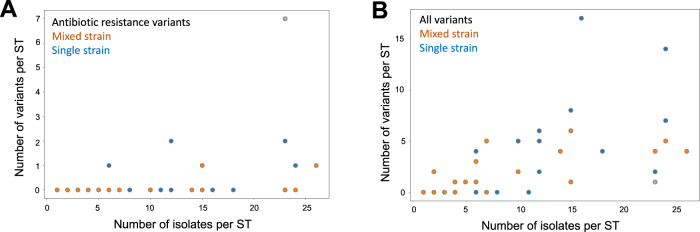
Genomic drivers of resistance evolution within patients. The abundance of variants in **A** genes associated with antibiotic resistance and **B** all variants as a function of sampling depth (i.e. number of isolates) in single strain (blue) and mixed strain (orange) patients. Each data point represents a unique combination of ST and patient. We modelled the number of variants with a negative binomial regression accounting for the infection type, number of isolates, and a possible interaction between them (n.variants ~ infection type*isolates). The number of isolates per ST was positively associated with increased number of resistance variants (0.13 +/− 0.05 variants per additional isolate; *z* = 2.57, *p* = 0.01) and other variants (0.13 +/− 0.03; *z* = 4.17, *p* < 0.001). There was no significant difference between STs from single strain and mixed strain patients in terms of **A** resistance variants or **B** genetic diversity (Supplementary Table 4a, 4b). We excluded a single strain population with an exceptionally high number of resistance variants (grey data point, see ‘Methods’), although including it did not change this conclusion (Supplementary Table 4c, 4d). The raw data is presented in the source data file.

To test for the mutational evolution of resistance, we searched for polymorphisms in genes that have been implicated in resistance using a database constructed by^45^. Because this approach only includes known resistance genes, it provides a conservative estimate of the presence of resistance genes. For example, the ability to predict resistance phenotypes based on known genomic resistance determinants is low for some antibiotics, such as meropenem, suggesting that important resistance genes remain to be discovered. We only considered within-host polymorphisms in this analysis under the assumption that these variants reflect de novo mutations that arose following patient colonization. We modelled the number of resistance polymorphisms for each patient/strain combination with a negative binomial regression as a function of the infection type (single-strain vs. mixed-strain) and the number of isolates sequenced to account for variation in sampling intensity. There was no significant difference in the number of de novo resistance variants between single-strain and mixed-strain populations (Fig. 4A, Supplementary Table 4a). As a control, we also measured levels of genome-wide polymorphism i.e. other variants not associated with resistance. Similarly, there was no significant effect on de novo variants from the infection type, providing good evidence that de novo variants arise at similar rates in both types of infection (Fig. 4B, Supplementary Table 4b).

Although studies of resistance in *Pseudomonas* have tended to focus on mutational resistance, there is growing evidence that *P. aeruginosa* acquires mobile resistance genes, including extended spectrum β-lactamases, carbapenemases, and aminoglycoside modifying enzymes^44,46^. We tested for the acquisition of new resistance genes through horizontal gene transfer by searching for increases in the prevalence of acquired resistance genes (again, using the database from ref. ^45^). We found no evidence for the acquisition of novel resistance suggesting that *Pseudomonas* acquires resistance genes at a low rate in the lung (see also ref. ^47^).

### Within-patient diversity and fitness trade-offs

Trade-offs between antibiotic resistance and fitness play a key role in the stability of resistance within patients following antibiotic treatment. Antibiotic treatment often fails to eradicate sensitive cells^8,34,48^, ensuring that competition between sensitive and resistant cells occurs following antibiotic treatment. When resistance is associated with low fitness due to trade-offs, competition can lead to the loss of resistance^8,34^, limiting the ability of the population to adapt to future antibiotic treatment^48^.

The acquisition of antibiotic resistance by mutation is typically associated with costs^49,50^, suggesting that trade-offs should be common in patients where resistance has evolved due to selection for de novo resistance mutations. Over time, resistant lineages can evolve to offset the fitness costs of resistance by acquiring compensatory mutations^51–54^. As such, we would expect trade-offs between resistance and fitness to be weaker when resistance evolves by selection for pre-existing resistant strains that have had more opportunity to acquire compensatory mutations.

The most commonly used method to test for fitness costs associated with resistance is to measure growth rate in antibiotic-free culture medium^49^. Culture medium does not replicate the physical and chemical complexity of host tissues and it lacks stressors that are likely to play an important role in vivo, such as host-derived antimicrobials. In spite of these clear limitations, estimates of growth rate measured in culture medium are moderately well correlated with in vivo estimates of fitness from animal models (*r* = 0.81; ref. ^49^), and there are compelling examples showing that in vitro growth rate can predict fitness of *P. aeruginosa* in critically ill patients from the ASPIRE-ICU study^8,34^.

**Fig. 5:**
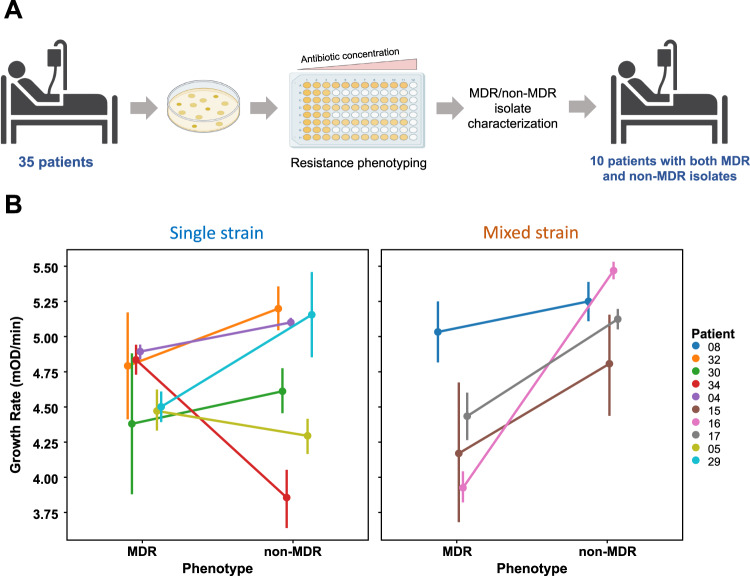
Fitness trade-offs within patients. **A** Selection of 10 patients with both MDR and non-MDR isolates from the total cohort of 35 patients based on resistance phenotyping data. **B** Points show the mean growth rate in antibiotic-free culture medium (+/−s.e.m.) of 179 MDR and non-MDR isolates from patients with single strain (*n* = 6) or mixed strain (*n* = 4) populations. Line colour corresponds to patient number as shown in key. MDR was associated with reduced growth rate (*P* = 0.021), and the trade-off associated with MDR was strongest in mixed strain populations (*P* = 0.001). The data from this assay was analysed using ANOVA (see ‘Methods’ for model details, and Supplementary Table 5 for model output) and the raw data are presented in the source data file.

To test for costs of resistance, we measured the growth rate of isolates from all patients containing a mixture of MDR and non-MDR *Pseudomonas* isolates (Fig. 5A; source data file). This subset of patients included both single-strain (*n* = 6) and mixed-strain patients (*n* = 4) (Fig. 5B). The MDR phenotype was associated with reduced growth, demonstrating a trade-off associated with resistance(main effect MDR: F_1,167_ = 5.42, *P* = 0.021) (Fig. 5B) (Supplementary Table 5). In contrast to our hypothesis, trade-offs were stronger in mixed strain populations than in single strain populations (MDR*diversity interaction: F_1,167_ = 11.24; *P* = 0.0010) (Fig. 5B) (Supplementary Table 5).

However, the genetic basis of this trade-off is not clear. Successful MDR/XDR strains of *P. aeruginosa* typically carry a suite of chromosomal resistance mutations and horizontally acquired resistance genes^38,55^ as opposed to the small number of SNPs in resistance genes found in patients colonized by a single strain, and this asymmetry in resistance gene content could explain the large costs associated with pre-existing resistant strains. Alternatively, the low fitness of pre-existing MDR strains could reflect fitness costs associated with other genes that are unrelated to antibiotic resistance per se.

## Discussion

Evolutionary approaches are increasingly being used to understand and combat antibiotic resistance^56–59^, and an important challenge for this field is to understand the within-host drivers of resistance^3–5,8,34^. The key finding of this study is that resistance evolves rapidly in patients colonized by diverse *P. aeruginosa* populations due to selection for pre-existing resistant strains, demonstrating a clear link between within-host diversity and resistance. Conventional methods used in clinical microbiology labs are systematically biased against the detection of pathogen diversity, making it difficult to assess the importance of pre-existing diversity in resistance across bacterial pathogens. The rate at which resistance evolves in patients varies widely across pathogens^60^, and we speculate that high levels of within-host diversity may explain why some pathogens, such as *Pseudomonas*, rapidly adapt to antibiotic treatment in patients^60^.

We also found evidence of mutations in genes that have previously been implicated in resistance, and an outstanding challenge is to determine if these polymorphisms were present as standing genetic variation at the onset of treatment (for example as a result of past antibiotic treatment) or if these polymorphisms arose as a result of spontaneous mutations during antibiotic treatment^48^. This could be done using amplicon sequencing to quantify the abundance of resistance polymorphisms before and after treatment in DNA extracted from patient samples, or by using phylogenetic approaches to date the origin of resistance lineages^8,34^. Recent work has used amplicon sequencing to show how within-host *P. aeruginosa* diversity is linked to lung disease progression in patients with cystic fibrosis^61^.

Trade-offs between resistance and growth rate make it is challenging to understand how strains that vary in resistance can stably coexist within the same patient over the long term. Therefore, we speculate that within-patient diversity will be high in settings with a high infection rate due to recurrent colonization^11,22,32^ or in patients where antibiotic exposure is variable across host tissues, allowing high and low resistance strains to coexist by occupying spatially distinct niches^62,63^. A further challenge will be to determine if mixed strain populations arise as a consequence of single colonization events or by superinfection^64^. For example, transmissible strains of *P. aeruginosa* can superinfect patients, giving rise to high within-patient strain diversity in CF patients^22,32,64,65^. Finally, it is possible that some medical conditions make it more likely for patients to be colonized by multiple strains due to variation in susceptibility to bacterial colonization.

In conclusion, our study underscores the importance of within-host bacterial diversity for understanding AMR^4,5,8,10,66,67^. Using bacterial isolates to measure within-host diversity limited the number of patients that we could include in this study and measuring bacterial diversity by sequencing DNA extracted directly from patient sample or from pools of isolates should make it possible to assess the link between diversity and AMR in larger cohorts of patients. Our findings suggest that measuring the diversity of pathogen populations might make it possible to more accurately predict the likelihood of treatment failure at the level of individual patients, in the same way that measurements of diversity in cancer cell populations have been informative for predicting the success of chemotherapy^68,69^.

## Methods

### Clinical data

The subjects were recruited as part of the observational, prospective, multicentre European epidemiological cohort study ASPIRE-ICU (The Advanced understanding of *Staphylococcus aureus* and *Pseudomonas aeruginosa* Infections in Europe–Intensive Care Units, (NCT02413242 ClinicalTrials.gov)^33^. This was conducted according to the principles of the Declaration of Helsinki, in accordance with the Medical Research Involving Human Subjects Act and local guidelines in the participating countries. The study protocol was approved by the research ethics committee in each country or participating hospitals. Written informed consent was obtained upon study enrolment from the participants or their legally accepted representative. Adult subjects were enroled between June 2015 and October 2018 within 3 days after ICU admission. To be eligible the patients needed to be on mechanical ventilation at ICU admission and have an expected length of hospital stay ≥48 h^33^. Participants were followed through their ICU stay to assess the development of pneumonia. Data on antibiotic use in the two weeks preceding ICU admission and during the ICU stay were reported. During ICU stay, lower respiratory tract samples were obtained three times in the first week, two times in the three following weeks, on the day of diagnosis of protocol pneumonia and seven days after it. The demographic and clinical baseline characteristics of the 35 subjects included in this analysis are given in Supplementary Table 6.

### Sample collection and isolation

Lower respiratory samples used in this study were collected within the ASPIRE-ICU study^33^. Untreated respiratory samples were stored at −80 °C until shipment and further analysis at the central lab at the University of Antwerp. The samples were blended (30,000 rpm, probe size 8 mm, steps of 10 s, max 60 s in total), diluted 1:1 v/v with Lysomucil (10% Acetylcysteine solution) (Zambon S.A, Belgium) and incubated for 30 min at 37 °C with 10 s vortexing every 15 min. Thereafter, quantitative culture was performed by inoculating 10-fold dilutions on CHROMID *P. aeruginosa* Agar and blood agar using spiral plater EddyJet (IUL, Spain). Plates were incubated at 37 °C for 24 h and CFU/mL was calculated. Plates without growth were further incubated for 48 h and 72 h. Matrix-Assisted Laser Desorption Ionization-Time of Flight Mass Spectrometry (MALDI-TOF MS) was used to identify up to 12 *P. aeruginosa* colonies per sample, which were stored at −80 °C until shipment to the University of Oxford and further use.

### Resistance phenotyping

All *P. aeruginosa* isolates were grown from glycerol stocks on Luria-Bertani (LB) Miller Agar plates overnight at 37 °C. Single colonies were then inoculated into LB Miller broth for 18–20 h overnight growth at 37 °C with shaking at 225 rpm. Overnight suspensions were serial diluted to ~5 × 10^5^ CFU/mL. Resistance phenotyping was carried out as minimum inhibitory concentration (MIC) testing via broth microdilution as defined by EUCAST recommendations^70,71^, with the alteration of LB Miller broth for growth media and the use of *P. aeruginosa* PAO1 as a reference strain. Antibiotics were assayed along the following 2-fold dilution series between: ciprofloxacin (0.125–16 µg/mL), aztreonam (1–128 µg/mL), ceftazidime (1–256 µg/mL), meropenem (0.25–64 µg/mL), piperacillin/tazobactam (2–256 µg/mL) and gentamicin (0.5–128 µg/mL). Growth inhibition was defined as OD_595_ < 0.200. We calculated a single biologically independent MIC for each of the 441 *P. aeruginosa* isolates on each antibiotic (Source Data). When an isolate reached the measurable limit of the MIC assay (i.e. not inhibited at the highest concentration used), the MIC was recorded as double of the upper limit in the raw data file of MIC results (Source data). The number of resistance phenotypes was calculated as the number of MICs per isolate above the following: ciprofloxacin (0.5 µg/mL), aztreonam (16 µg/mL), ceftazidime (8 µg/mL), meropenem (8 µg/mL), piperacillin/tazobactam (16 µg/mL) and gentamicin (8 µg/mL). These points were set from EUCAST guidelines for *Pseudomonas* (v11 breakpoint table, and MIC distributions for *P. aeruginosa* data for gentamicin)^71^. MDR isolates were defined as isolates with 3 or more resistance phenotypes. For longitudinally sampled patients treated with colistin (patient 6, patient 16, patient 18, patient 32, patient 35), the same protocol was used to determine colistin MIC along the following 2-fold dilution series between: 0.5–64 µg/mL, and a colistin resistance phenotype was determined as an MIC above 2 µg/mL (Source data)^71^.

### Sequencing

All isolates were sequenced in the MiSeq or NextSeq illumina platforms yielding a sequencing coverage of 21X–142X. Raw reads were quality controlled with the ILLUMINACLIP (2:30:10) and SLIDINGWINDOW (4:15) in trimmomatic v. 0.39. Quality controlled reads were assembled for each isolate with SPAdes v. 3.13.1 with default parameters. These assemblies were further polished using pilon v. 1.23 with minimum number of flank bases of 10, gap margin of 100,000, and kmer size of 47. Resulting contigs were annotated based on the P. aeruginosa strain UCBPP-PA14 in prokka v. 1.14.0. Each isolate was typed using the *Pseudomonas aeruginosa* multi-locus sequence typing (MLST) scheme from PubMLST (Last accessed on 11.06.2021). Sixteen isolates chosen randomly from the key STs were sequenced with the Oxford nanopore MinION platform using the FLO-MIN106 flow-cell and the SQK-LSK109 kit. Raw reads were basecalled using guppy v. 0.0.0 + 7969d57 and reads were demultiplexed using qcat v. 1.1.0 (https://github.com/nanoporetech/qcat). Resulting sequencing reads were assembled using unicycler v. 0.4.8^72^, which used SAMtools v. 1.9^73^, pilon v. 1.23^74^, and bowtie2 v. 2.3.5.1^75^, in hybrid mode with respective illumina reads.

For the rarefaction analysis of mixed strain patient samples the datasets were subsampled 100X from size 2 to N-1, where N is the actual size of the sample, using the standard random library of python v. 3.9.16. The null hypothesis that the sampling is normally distributed was tested using the normaltest function from the script library^76,77^.

### Variant calling

Paired-ended reads were mapped to the *P. aeruginosa* PAO1 reference genome (NC_002516.2) with Bowtie 2 v2.2.4, and pileup and raw files were obtained by using SAMtools v0.1.16 and PicardTools v1.140, using the Genome Analysis Toolkit (GATK) v3.4-46 for realignment around InDels. From the raw files, SNPs were extracted if they met the following criteria: a quality score (Phred-scaled probability of the samples reads being homozygous reference) of at least 50, a root-mean-square (RMS) mapping quality of at least 25 and a coverage depth of at least 3 reads, excluding all ambiguous variants. MicroInDels were extracted from the totalpileup files when meeting the following criteria: a quality score of at least 500, a RMS mapping quality of at least 25 and support from at least one-fifth of the covering reads^78^. Filtered files were converted to vcf and SNPs and InDels were annotated with SnpEff v4.2.^79^. Gene absence was evaluated using SeqMonk (https://www.bioinformatics.babraham.ac.uk/projects/seqmonk/) and OprD structural integrity was investigated within the de novo assemblies using an appropriate reference sequence. Finally, all mutations within a set of genes known to be involved in antibiotic resistance were extracted and natural occurring polymorphisms were filtered^45^. The presence of horizontally acquired antimicrobial resistance determinants was also investigated using the web tool ResFinder (https://cge.cbs.dtu.dk/services/ResFinder/).

To identify mutations and gene gain/loss during the infection, short-length sequencing reads from each isolate were mapped to each of the four long-read de novo assemblies with bwa v. 0.7.17 using the BWA-MEM algorithm. Preliminary SNPs were identified with SAMtools and BCFtools v. 1.9. Low-quality SNPs were filtered out using a two-step SNP calling pipeline, which first identified potential SNPs using the following criteria: (1) Variant Phred quality score of 30 or higher, (2) At least 150 bases away from contig edge or indel, and (3) 20 or more sequencing reads covering the potential SNP position. In the second step, each preliminary SNP was reviewed for evidence of support for the reference or the variant base; at least 80% of reads of Phred quality score of 25 or higher were required to support the final call. An ambiguous call was defined as one with not enough support for the reference or the variant, and, in total, only one non-phylogenetically informative SNP position had ambiguous calls. Indels were identified by the overlap between the HaplotypeCaller of GATK v. 4.1.3.0 and breseq v. 0.34.0. The variable genome was surveyed using GenAPI v. 1.098 based on the prokka annotation of the short-read de novo assemblies. The presence or absence of genes in the potential variable genome was reviewed by mapping the sequencing reads to the respective genes with BWA v.0.7.17.SNPs/indels. A maximum parsimony phylogeny was constructed based on high-confidence SNPs to illustrate the genetic diversity of isolates recovered from patient 15 (Supplementary Fig. 2). Phylogenies were plotted with iTOL^80^.

### Growth assays

All isolates were grown from glycerol stocks on LB Miller Agar plates overnight at 37 °C. Single colonies were then inoculated into LB Miller broth for 18–20 h overnight growth at 37 °C with shaking at 225 rpm. Overnight suspensions were serially diluted to an OD_595_ of ~0.05 and placed within the inner 60 wells of a 96-well plate equipped with a lid. To calculate growth rate, isolates were then grown in LB Miller broth at 37 °C and optical density (OD595nm) measurements were taken at 10-min intervals in a BioTek Synergy 2 microplate reader set to moderate continuous shaking. Growth rate was then calculated as the maximum slope of OD versus time over an interval of ten consecutive readings, and at least three biologically independent replicate cultures were measured for all of the pulmonary isolates to calculate the mean growth rate of each isolate (Source data).

### Statistics

For each patient sample, we calculated the proportion of isolates that were resistant to each antibiotic as described above^70,71^. Changes in resistance for each antibiotic were measured as the difference in proportion of resistant isolates between the final and initial sample for longitudinally sampled patients (Source data), giving a total of 6 responses per patient (i.e. 1 response per antibiotic/patient combination). Although the proportions of isolates that were resistant to antibiotics were not normally distributed, changes in the proportion of resistant isolates were normally distributed. To test drivers of antibiotic resistance we used an ANOVA that included main effects of initial proportion of resistant isolates (continuous variable, 1DF), antibiotic (5 DF), pathogen diversity (single strain or mixed strain; 1DF) and response type (direct or collateral, 1DF. We nested patient within pathogen diversity (11DF). We included an interactions between initial proportion of resistant isolates and pathogen diversity (1DF) and initial resistance and antibiotic (1DF). Non-significant terms were then sequentially removed to yield a reduced model containing only significant effects (Supplementary Table 3).

The number of observed de novo variants for each ST/patient combination (n.variants) can be treated as a count variable. Because of overdispersion we used a negative binomial regression using the glm.nb function in the “MASS” R package v7.3-55^81^. We controlled for single vs. mixed infections (infection.type) and number of sequenced isolates per ST (isolates.per.ST) using the model formula: n.variants ~ infection.type*isolates.per.ST. We ran separate models for resistance-associated variants (Supplementary Table 4a) and all other variants (Supplementary Table 4b). In both models, the only significant association was with the number of sequenced isolates (*p* < 0.05 for both). This analysis excluded one outlier from a single-strain patient (isolate 25-0925) where we found many polymorphic deletions in genes associated with antibiotic efflux. However, including the outlier did not change the conclusion that infection type had no significant effect (Supplementary Table 4c, 4d).

Fitness costs were assessed by comparing the growth rate of co-occurring MDR (i.e. 3 or more resistance phenotypes) and non-MDR (i.e. 0-2 resistance phenotypes) lung isolates from the same patient. We considered all patients with multiple (i.e. >1) MDR and non-MDR isolates in this analysis, giving a total of 179 isolates from 10 patients (Source data). To understand the sources of variation in fitness, we used an ANOVA that included main effects of resistance phenotype (i.e. MDR or non-MDR; 1DF), pathogen diversity (single strain or mixed strain; 1DF) and patient nested within pathogen diversity (8DF). We tested for variation in fitness trade-offs between single strain and mixed strain populations by including a resistance phenotype*pathogen diversity interaction in the model. The full statistical model is presented in Supplementary Table 5. JMP v.12 was used for statistical analysis.

### Reporting summary

Further information on research design is available in the Nature Portfolio Reporting Summary linked to this article.

## Supplementary information

Supplementary Information

Reporting Summary

## Data Availability

The source data have been deposited in the Oxford Research Archive for Data (10.5287/ora-mzzd1qykn). Isolates can be obtained from the corresponding author for research use via an MTA subject to permission from the ASPIRE research committee. All sequencing data generated in this study and all isolate assemblies can be found at: (https://www.ncbi.nlm.nih.gov/bioproject/PRJNA974969). All clinical data analysed for these patients as part of this study are included in this article within the supplementary information or source data file. Source data are provided with this paper.

## Source data

Source Data
