# Supplementary material for: Mixed strain pathogen populations accelerate the evolution of antibiotic resistance in patients
Source: Nat Commun. 2023 Jul 12;14:4083. doi: 10.1038/s41467-023-39416-2 (PMC10338428; doi:10.1038/s41467-023-39416-2)
Supplement: Supplementary file 1 — Supplementary Information [file 41467_2023_39416_MOESM1_ESM.pdf]

Supplementary Materials for

Mixed strain pathogen populations accelerate the evolution of antibiotic resistance in patients

Supplementary Table 1. Comparison of *P. aeruginosa* positivity in first enrolment sample between single and mixed strain patients.

Patient	Mixed or single strain?	First enrolment sample *P. aeruginosa* positive?
1	Single strain	Negative
3	Single strain	Positive
4	Single strain	Positive
5	Single strain	Positive
6	Single strain	Positive
7	Single strain	Positive
12	Single strain	Positive
13	Single strain	Negative
19	Single strain	Negative
21	Single strain	Negative
22	Single strain	Negative
23	Single strain	Negative
24	Single strain	Negative
25	Single strain	Negative
26	Single strain	Negative
27	Single strain	Positive
29	Single strain	Positive
30	Single strain	Positive
31	Single strain	Negative
32	Single strain	Positive
33	Single strain	Positive
34	Single strain	Negative
35	Single strain	Positive
2	Mixed strain	Negative
8	Mixed strain	Positive
9	Mixed strain	Negative
10	Mixed strain	Positive
11	Mixed strain	Negative
14	Mixed strain	Negative
15	Mixed strain	Negative
16	Mixed strain	Positive
17	Mixed strain	Negative
18	Mixed strain	Positive
20	Mixed strain	Positive
28	Mixed strain	Positive

The first enrolment was *P. aeruginosa* positive in a total of 12/23 (52.17%) single strain patients and 6/12 (50.00%) mixed strain patients. Whether first enrolment sample was *P. aeruginosa* positive did not differ significantly between single strain and mixed strain patients (unpaired two tailed t-test; p=0.9073).

Supplementary Table 2: Time (days) between first and last sample for antibiotic-treated single strain and mixed strain patients.

Patient number	Mixed or single strain?	Time (days) between first and last sample
8	Mixed strain	4
15	Mixed strain	7
17	Mixed strain	4
10	Mixed strain	7
16	Mixed strain	20
18	Mixed strain	16
29	Single strain	7
32	Single strain	4
34	Single strain	4
6	Single strain	21
35	Single strain	22
5	Single strain	30
4	Single strain	14

Time (days) between first and last sample did not differ significantly between antibiotic-treated single and mixed strain patients (unpaired two tailed t-test; p=0.3211).

Supplementary Table 3: ANOVA testing responses to antibiotic treatment.

Response variable: $P_{R(F),x}$- $P_{R(I),x}$ where $P_{R(F),x}$ is the proportion of isolates resistant from patient y to antibiotic *x* at the final time point and $P_{R(I),x}$ is the proportion of isolates from patient y resistant to antibiotic *x* at the initial time point

Pathogen diversity: A categorical variable reflecting patients that were colonized by single strain and mixed strain populations.

Patient: We nested patient within pathogen diversity as patients were colonized by either a single strain or mixed strain population of *Pseudomonas*.

Antibiotic: Resistance was measured to six different antibiotics (meropenem, ceftazidime, gentamicin, ciprofloxacin, aztreonam, tazobactam)

Initial Resistance: $P_{R(I),x}$ is the proportion of isolates from patient y resistant to antibiotic *x* at the initial time point

Response type: A binary variable reflecting whether or not antibiotics were used to treat a given patient during the sampling interval (ie direct responses) or not (collateral responses)

A) Full model

Term	DF	SS	F	P
Pathogen diversity	1	8456	15.03	.0003
Patient [Pathogen diversity]	11	24244	3.91	.0004
Antibiotic	5	2391	.85	.52
Initial resistance	1	17391	30.9	<.0001
Response type	1	16.13	.029	.866
Initial resistance*pathogen diversity	1	9.98	.0177	.894
Initial resistance*Antibiotic	5	2515	.894	.49
Error	52	29253		

B) Reduced model – constructed by sequentially removing non-significant terms, beginning with interaction terms.

Term	DF	SS	F	P
Pathogen diversity	1	8393	15.98	.0002
Patient [Pathogen diversity]	11	28919	5.00	<.0001
Initial resistance	1	17180	32.70	<.0001
Error	64	33626		

C) Full model excluding the data from patient 8

Term	DF	SS	F	P
Pathogen diversity	1	6505	12.17	.0011
Patient [Pathogen diversity]	10	24365	4.55	.0002
Antibiotic	5	1727	.64	.66
Initial resistance	1	16910	31.6	<.0001
Response type	1	24.47	.045	.83
Initial resistance*pathogen diversity	1	24.76	.046	.83
Initial resistance*Antibiotic	5	4202	1.57	.18
Error	47	25121		

Supplementary Table 4: Summary of negative binomial regression models for the number of *de novo* variants detected. Coefficients and p-values (two-sided Wald test) are given for resistance variants (a) and other variants (b), including rerunning analysis with an outlier which did not change the overall conclusion (c, d).

**(a) glm.nb(resistance.variants ~ infection.type*isolate.count)**	Estimate	Std. Error	z value	Pr(>|z|)
(Intercept)	-2.8715501	0.95946467	-2.9928669	0.0027637
Infection.type (mixed vs. single)	-2.2690002	2.28450297	-0.9932139	0.32060572
Isolate.count (no. of isolates)	0.13180623	0.05133518	2.56756165	0.01024166
Interaction between infection type and no. of isolates	0.04884186	0.10804483	0.45205175	0.6512317

**(b) glm.nb(nonresistance.variants ~ infection.type*isolate.count)**	Estimate	Std. Error	z value	Pr(>|z|)
(Intercept)	-0.420218	0.44753492	-0.9389613	0.34775063
Infection.type (mixed vs. single)	-0.5238572	0.57749448	-0.9071206	0.364343
Isolate.count (no. of isolates)	0.12915218	0.03094087	4.17416147	2.99E-05
Interaction between infection type and no. of isolates	-0.00666	0.04064429	-0.16386	0.86984136

**(c) With 25-0925 outlier: glm.nb(resistance.variants ~ infection.type*isolate.count)**	Estimate	Std. Error	z value	Pr(>|z|)
(Intercept)	-3.3612227	1.02884311	-3.2669925	0.00108697
Infection.type (mixed vs. single)	-1.7874635	2.33750836	-0.7646876	0.44445762
Infection.type (mixed vs. single)	0.1834749	0.0534047	3.43555715	0.00059134
Interaction between infection type and no. of isolates	-0.0016854	0.11209265	-0.0150359	0.98800352

**(d) With 25-0925 outlier: glm.nb(nonresistance.variants ~ infection.type*isolate.count)**	Estimate	Std. Error	z value	Pr(>|z|)
(Intercept)	-0.2828904	0.45670829	-0.6194117	0.5356452
Infection.type (mixed vs. single)	-0.6852173	0.59202829	-1.1574063	0.24710641
Isolate.count (no. of isolates)	0.11354428	0.03077403	3.68961384	0.00022459
Interaction between infection type and no. of isolates	0.01143137	0.04144573	0.27581529	0.78268994

Supplementary Table 5: ANOVA testing for a trade-off between growth rate and resistance.

Response variable: Growth rate, the mean growth rate of 8-10 (check) replicate cultures of each isolate in culture medium

Pathogen diversity: A categorical variable reflecting isolates from patients that were colonized by single strain or mixed strain populations.

Resistance phenotype: A categorical variable reflecting isolates that were MDR (resistant to 3 or more antibiotics) or non-MDR (resistant to 0-2 antibiotics)

Patient: We nested patient within pathogen diversity as patients were colonized by either a single strain or mixed strain population of Pseudomonas.

**Term**	**DF**	**SS**	**F**	**P**
Pathogen diversity	1	.062	.126	.72
Resistance phenotype	1	2.66	5.42	.021
Pathogen diversity*Resistance phenotype	1	5.52	11.24	.0010
Patient [Pathogen diversity]	8	14.4	3.66	.0006
Error	167	82		

Supplementary Table 6: Characteristics of study cohort.

		**Included, n (%) (n = 35)**
Female sex		13 (36.1)
Age	(median, IQR)	63 (49-72)
APACHE IV score at ICU admission	(median, IQR)	45 (34-68)
Body Mass Index, BMI	(median, IQR)	23 (26-31)
ICU admission specialty	Medical	15 (42.9)
	Surgery/trauma	20 (57.1)
Origin prior to ICU	Home/community	16 (45.7)
	Healthcare	19 (54.3)
Prior antibiotic use		10 (27.8)
Diabetes		6 (17.1)
European region	North	3 (8.6)
	South	24 (68.6)
	East	3 (8.6)
	West	5 (14.3)

Supplementary Figure 1: Rarefaction analysis of mixed strain patient samples, showing the proportion of subsamples that contain at least 2 strains as sample size increase.

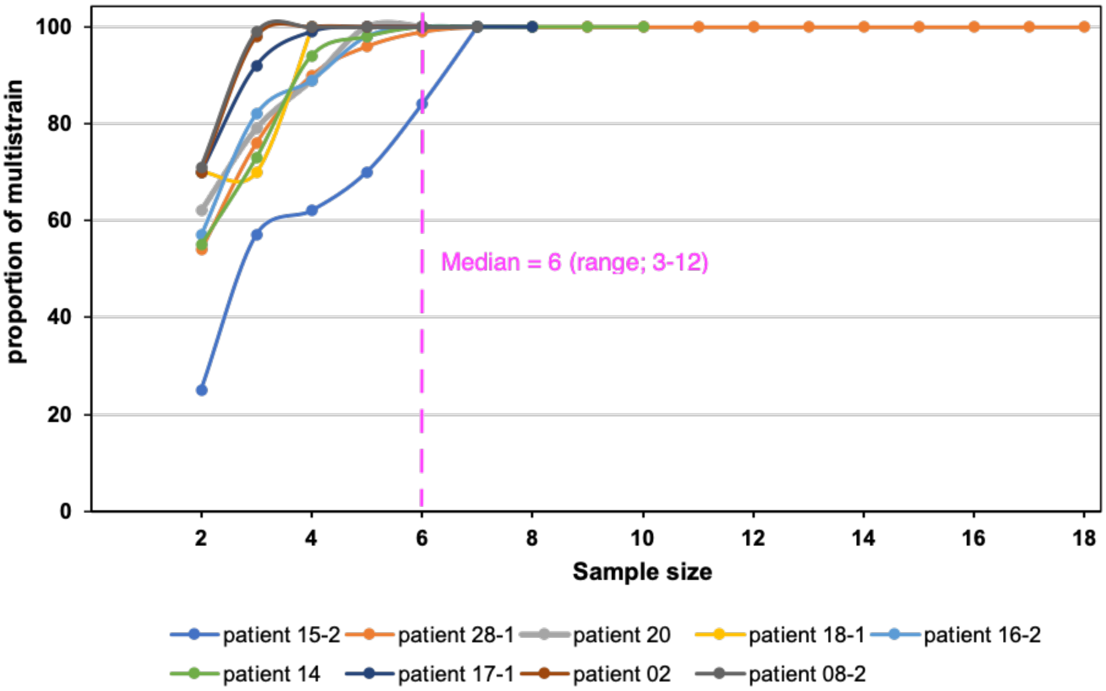

The median sample size of the single strain patient samples was 6 isolates, with a range between 3 and 12.

Supplementary Figure 2: Genetic diversity of isolates recovered from patient 15.

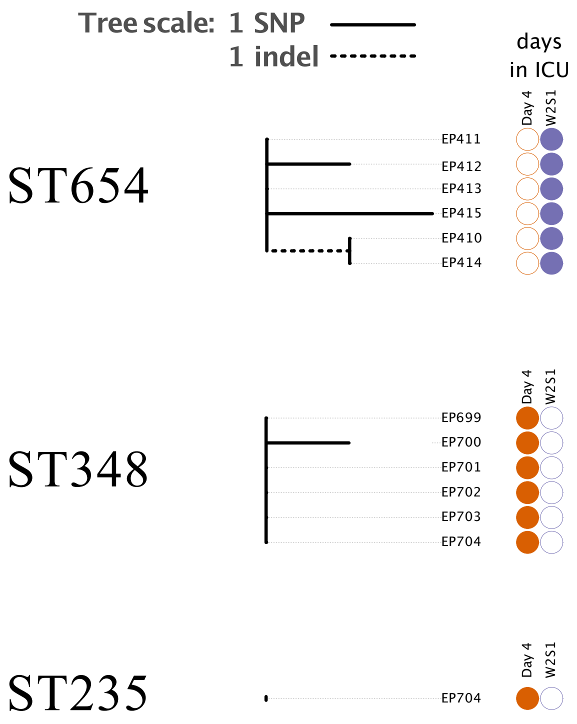

Phylogenetic reconstruction of ST654, ST348 and ST235 isolates from patient 15 indicating SNPs and indels.
