# Supplementary material for: Mixed strain pathogen populations accelerate the evolution of antibiotic resistance in patients
Source: Nat Commun. 2023 Jul 12;14:4083. doi: 10.1038/s41467-023-39416-2 (PMC10338428; doi:10.1038/s41467-023-39416-2)
Supplement: Supplementary file 2 — Reporting Summary [file 41467_2023_39416_MOESM2_ESM.pdf]

nature portfolio

Corresponding author(s): Craig MacLean

Last updated by author(s): May 22, 2023

Reporting Summary

Nature Portfolio wishes to improve the reproducibility of the work that we publish. This form provides structure for consistency and transparency in reporting. For further information on Nature Portfolio policies, see our Editorial Policies and the Editorial Policy Checklist.

Statistics

For all statistical analyses, confirm that the following items are present in the figure legend, table legend, main text, or Methods section.

n/a	Confirmed	
☐	☒	The exact sample size (*n*) for each experimental group/condition, given as a discrete number and unit of measurement
☐	☒	A statement on whether measurements were taken from distinct samples or whether the same sample was measured repeatedly
☐	☒	The statistical test(s) used AND whether they are one- or two-sided<br>*Only common tests should be described solely by name; describe more complex techniques in the Methods section.*
☒	☐	A description of all covariates tested
☒	☐	A description of any assumptions or corrections, such as tests of normality and adjustment for multiple comparisons
☐	☒	A full description of the statistical parameters including central tendency (e.g. means) or other basic estimates (e.g. regression coefficient) AND variation (e.g. standard deviation) or associated estimates of uncertainty (e.g. confidence intervals)
☐	☒	For null hypothesis testing, the test statistic (e.g. *F*, *t*, *r*) with confidence intervals, effect sizes, degrees of freedom and *P* value noted<br>*Give P values as exact values whenever suitable.*
☒	☐	For Bayesian analysis, information on the choice of priors and Markov chain Monte Carlo settings
☒	☐	For hierarchical and complex designs, identification of the appropriate level for tests and full reporting of outcomes
☐	☒	Estimates of effect sizes (e.g. Cohen's *d*, Pearson's *r*), indicating how they were calculated

Our web collection on statistics for biologists contains articles on many of the points above.

Software and code

Policy information about availability of computer code

Data collection	Data was entered in a web-based electronic data capture system that was designed for ASPIRE-ICU. The study site entered data in the system from the subject's source documents (i.e. medical chart).
Data analysis	JMP v.12 was used for statistical analysis. Trimmomatic v. 0.39, SPAdes v. 3.13.1, pilon v. 1.23, prokka v. 1.14.0, qcat v. 1.1.0, and unicycler v. 0.4.8 were used for sequencing data assembly and annotation. Bowtie 2 v2.2.4, SAMtools v0.1.16, PicardTools v1.140, BWA v. 0.7.17, BCFtools v. 1.9, HaplotypeCaller of GATK v. 4.1.3.0, SnpEff v4.2, GenAPI v. 1.098, and breseq v. 0.34.0 were used for variant calling.

For manuscripts utilizing custom algorithms or software that are central to the research but not yet described in published literature, software must be made available to editors and reviewers. We strongly encourage code deposition in a community repository (e.g. GitHub). See the Nature Portfolio guidelines for submitting code & software for further information.

Data

Policy information about availability of data

All manuscripts must include a data availability statement. This statement should provide the following information, where applicable:

- Accession codes, unique identifiers, or web links for publicly available datasets
- A description of any restrictions on data availability
- For clinical datasets or third party data, please ensure that the statement adheres to our policy

- Source data is available with the paper and has additionally been deposited in the Oxford Research Archive for Data (DOI: 10.5287/ora-mzzd1qykn)

- Clinical data analyzed for this patient as part of the study are included in this article. Further clinical data are not publicly available for confidentiality reasons but are available upon scientific review and approval of request by the Study's Scienctific Committee.
- Isolates can be obtained from the corresponding author for research use via an MTA subject to permission from the ASPIRE research committee.
- Publicly available datasets used in study include PubMLST (last accessed on 11.06.2021), and the Pseudomonas aeruginosa PAO1 reference genome (NC_002516.2)
- All sequence data and isolate assemblies have been deposited in a publicly available database as a bioproject (https://www.ncbi.nlm.nih.gov/bioproject/PRJNA974969).

Human research participants

Policy information about studies involving human research participants and Sex and Gender in Research.

Reporting on sex and gender	13/35 patients were of female sex and 22/35 patients were of male sex, gender was not reported. The small sample size did not allow for stratification based on sex.
Population characteristics	The median age of the 35 patients included in this study was 63 years (interquartile range [IQR] 49-72). 13/35 patients were of female sex. The median APACHE IV score at intensive care unit (ICU) admission was 45 (IQR 34-68), while the median Body Mass Index was 23 (IQR 26-31).The ICU admission specialty was medical in 15 (42.9%) cases, and surgical/trauma in 20 (57.1%) cases. Regarding the origin prior to ICU admission, 16 (45.7%) patients were coming from the community, whereas 19 (54.3%) were transferred from other healthcare institutions. The majority of patients (68.6%) were enrolled in countries in Southern Europe.
Recruitment	All consecutive adult ICU patients fulfilling inclusion criteria were approached for participation in the ASPIRE-ICU study (full details of ASPIRE-ICU study are publicly available here: https://www.ncbi.nlm.nih.gov/pmc/articles/PMC5613521/).<br>The study is composed of two study populations, the surveillance population and study cohort population. The study cohort is nested within the larger surveillance population; this means that all data and specimens collected specifically for study cohort participants is in addition to data already captured by ways of surveillance. Patients eligible to participate in the surveillance population must be on mechanical ventilation (MV) upon or (expected to be) within 24 h after ICU admission and have an expected length of stay (LOS) of at least 48 h. Patients with an expected ICU stay of less than 48 h are at a lower risk for developing ICU infections since this population is generally healthier, without significant comorbidities and shorter in the ICU. Surveillance patients that meet the eligibility criteria described below for the study cohort population will be enrolled. 2000 study cohort subjects are required to meet the objectives of this study.<br>Inclusion criteria for study cohort<br>-Participant is 18 years or older at the time of enrollment.<br>-Participant is on mechanical ventilation at ICU admission, or is (expected to be) within 24 h thereafter, based on investigator’s judgment.<br>-Expected stay in ICU is 48 h or longer based on investigator’s judgment.<br>-S. aureus colonization status is known within 72 h after start of first episode of mechanical ventilation and according to the result, the patient qualifies for enrollment.<br>-Written informed consent from subject / legally accepted representative within 72 h after start of first episode of mechanical ventilation.<br><br>Exclusion criteria for study cohort<br>-Previous participation as a subject in the study cohort of this study.<br>-Simultaneous participation of the subject in any preventive experimental study into anti-staphylococcus or anti-pseudomonas aeruginosa interventions.<br>-Expected death (moribund status) within 48 h, or ICU discharge of the participant within 24 h, at the moment of informed consent.<br><br>The subjects described in this study were study cohort participants for which multiple isolates per timepoint were collected and shipped to Oxford for analysis by the end of 2019.
Ethics oversight	The subjects were recruited as part of the observational, prospective, multicentre European epidemiological cohort study ASPIRE-ICU (The Advanced understanding of Staphylococcus aureus and Pseudomonas aeruginosa Infections in Europe–Intensive Care Units, (NCT02413242 ClinicalTrials.gov). This was conducted according to the principles of the Declaration of Helsinki, in accordance with the Medical Research Involving Human Subjects Act and local guidelines in the participating countries. The study protocol was approved by the research ethics committee in each country or participating hospitals. Written informed consent was obtained upon study enrolment from the participants or their legally accepted representative.

Note that full information on the approval of the study protocol must also be provided in the manuscript.

Field-specific reporting

Please select the one below that is the best fit for your research. If you are not sure, read the appropriate sections before making your selection.

☐ Life sciences ☐ Behavioural & social sciences ☒ Ecological, evolutionary & environmental sciences

For a reference copy of the document with all sections, see nature.com/documents/nr-reporting-summary-flat.pdf

Ecological, evolutionary & environmental sciences study design

All studies must disclose on these points even when the disclosure is negative.

Study description	In this study we assess the link between bacterial diversity and antibiotic resistance in a cohort of patients from the ASPIRE-ICU study.
Research sample	The research sample was a collection of 441 isolates that were collected from a total of 35 patients. Inclusion criteria for study cohort is given above in the human participants section. The subjects included in this study were study cohort participants for which multiple isolates per timepoint were sampled, confirmed as Pseudomonas aeruginosa via MALDI-TOF, and shipped to Oxford for analysis by the end of 2019. The samples were obtained from the lower respiratory tracts of patients, and so are meant to represent the Pseudomonas population present in patient lungs.
Sampling strategy	Isolates were randomly chosen from patient samples that tested positive for Pseudomonas aeruginosa. The protocol for the ASPIRE-ICU trial was to randomly collect 12 isolates per patient sample. However, in some cases our sample number of isolates was less than 12. This involved cases where (i) the total number of isolates recovered from a patient sample was <12 or (ii) when sequencing revealed that isolates were contaminated by other bacterial species. This sample size of 12 isolates per patients were chosen as the upper limit of feasibility of collection, to maximize patient number inclusion and depth of single sample analysis. Lower respiratory tract samples were collected on the following time intervals: day 1 (day of informed consent), day 4, day 7, and then twice weekly for 30 days or until ICU discharge. For patients not diagnosed with pneumonia, bacterial isolates were only collected from the day 1 and day 30 samples.
Data collection	Clinical data was recorded by staff at the participating hospitals. Data was entered in a web-based electronic data capture system that was designed for ASPIRE-ICU. The study site entered data in the system from the subject's source documents (i.e. medical chart). Data from isolate characterisation was collected on a BioTek Synergy 2 microplate reader and exported to Microscoft Excel.
Timing and spatial scale	Lower respiratory tract samples were collected on the following time intervals: day 1 (day of informed consent), day 4, day 7, and then twice weekly for 30 days or until ICU discharge. For patients not diagnosed with pneumonia, bacterial isolates were only collected from the day 1 and day 30 samples. A single endotracheal aspirate sample was taken at each time point, and we have assumed that these are representative of the lung population.
Data exclusions	We excluded data from a single patient for our analysis of within-patient genetic diversity (Figure 4) as this patient was clearly a statistical outlier. We repeated all analyses including this patient, and this did not change the conclusions of our analyses. No further data was excluded from the analysis.
Reproducibility	All experiments were replicated, as outlined in the methods. No attempts to carry out experiments failed. A single independent MIC was calculated for each Pseudomonas isolate on each antibiotic, and the combination of six antibiotic MIC scores were used to determine resistance phenotype for each isolate. At least 3 biologically independent replicates were used to determine growth rates for each isolate.
Randomization	The clinical trial was observational, meaning that patient treatment was not manipulated as part of the trial. We were able to allocate patients into groups based on the antibiotics that they received as part of their normal treatment (ie treated/untreated) or as a function of the level of genetic diversity found in the isolates (ie single strain or mixed strain).
Blinding	All phenotypic assays were blinded, in the sense that it was not known at the time of the assay which patient group the isolates being analysed were taken from.

Did the study involve field work? ☐ Yes ☒ No

Reporting for specific materials, systems and methods

We require information from authors about some types of materials, experimental systems and methods used in many studies. Here, indicate whether each material, system or method listed is relevant to your study. If you are not sure if a list item applies to your research, read the appropriate section before selecting a response.

Materials & experimental systems

n/a	Involved in the study	
☒	☐	Antibodies
☒	☐	Eukaryotic cell lines
☒	☐	Palaeontology and archaeology
☒	☐	Animals and other organisms
☐	☒	Clinical data
☒	☐	Dual use research of concern

Methods

n/a	Involved in the study	
☒	☐	ChIP-seq
☒	☐	Flow cytometry
☒	☐	MRI-based neuroimaging

Clinical data

Policy information about clinical studies

All manuscripts should comply with the ICMJE guidelines for publication of clinical research and a completed CONSORT checklist must be included with all submissions.

Clinical trial registration	ASPIRE-ICU ClinicalTrials.gov Identifier: NCT02413242
Study protocol	Paling, F.P., et al., Rationale and design of ASPIRE-ICU: a prospective cohort study on the incidence and predictors of Staphylococcus aureus and Pseudomonas aeruginosa pneumonia in the ICU. BMC infectious diseases, 2017. 17(1): p. 643 (the full ASPIRE-ICU protocol can be obtained upon request).
Data collection	ASPIRE-ICU was a cohort study of adult ICU patients at 30 hospitals in 11 European countries that recruited participants between June 2015 and October 2018.
Outcomes	The primary outcome (Pseudomonas aeruginosa pneumonia acquired in the ICU) was assessed in multiple steps. First, the following 4 clinical criteria were assessed daily: any new antibiotic use, new blood cultures performed, new chest radiograph or computed tomography scan that shows a new or worsening infiltrate, or other new reason to suspect pneumonia. In cases of at positive answer, a combination of objective major and minor criteria was assessed to categorize patients as having protocol-defined pneumonia or not, as described elsewhere. The primary end point was determined post hoc on the basis of isolation of P. aeruginosa from any lower respiratory tract specimen (including both clinical and study surveillance cultures) or blood culture in the 3 days before and after the day of pneumonia diagnosis, according to clinical criteria. Death was assessed at ICU discharge, at day 30 and day 90 after ICU admission. No other secondary outcomes were evaluated. These outcomes are not relevant to the work described in this study, where we analysed all patients who met the study inclusion criteria and had Pseudomonas aeruginosa isolates sampled during their ICU stay, regardless of pneumonia diagnosis status.
